# Validation of Spanish Language Evaluation Instruments for Body Dysmorphic Disorder and the Dysmorphic Concern Construct

**DOI:** 10.3389/fpsyg.2017.01107

**Published:** 2017-06-30

**Authors:** Cristina Senín-Calderón, María Valdés-Díaz, Ma M. Benítez-Hernández, Ma C. Núñez-Gaitán, Salvador Perona-Garcelán, Rafael Martínez-Cervantes, Juan F. Rodríguez-Testal

**Affiliations:** ^1^Department of Psychology, University of CádizCádiz, Spain; ^2^Personality, Evaluation and Psychological Treatment Department, University of SevilleSeville, Spain; ^3^Virgen del Rocío Outpatient Mental Hospital, Andalusian Health-CareSeville, Spain

**Keywords:** dysmorphic concern, DCQ, BDDE-SR, risk, Body Dysmorphic Disorder

## Abstract

Dysmorphic concern (DC) refers to excessive preoccupation with a slight or imagined defect in physical appearance with social avoidance and behavior directed at controlling the defect in appearance. This study attempted to adapt the factor structure of two instruments that cover the DC construct, the Dysmorphic Concern Questionnaire (DCQ) and the Body Dysmorphic Disorder Examination Self-Report (BDDE-SR), to Spanish and establish their psychometric properties. A total of 920 subjects (62.7% women, *M*_age_ = 32.44 years) participated. Exploratory and Confirmatory Factor Analysis of both scales found adequate goodness of fit indices. A one-dimensional structure was found for the DCQ and two first-order factors (dissatisfaction/preoccupation with body image (BI) and BI avoidance behavior) were identified for the BDDE-SR. The psychometric test–retest reliability and validity properties (content, convergent, and discriminant) were satisfactory. It is suggested that the DC construct includes both cognitive and behavioral aspects and may represent a continuum of severity with Body Dysmorphic Disorder at the end.

## Introduction

The study of alterations in body image (BI) is justified in a Western society where physical appearance is considered an indicator of social and professional success. An ideal of beauty (thin, young, sculpted, or worked on bodies, etc.) is transferred by communications media, by peers, family or partner, and sinks into the self-consciousness of people in general and the youngest in particular (Rodríguez-Testal, 2013).

Alterations in BI include other diagnostic expressions apart from Eating Disorders, especially Body Dysmorphic Disorder (BDD) (Cash and Smolak, 2011; Rodríguez-Testal, 2013). BDD is a preoccupation with barely perceptible or imagined flaws in physical appearance believed to be deformed or unattractive. Preoccupation means spending long times thinking about the perceived defect. BDD also involves difficulty in controlling/resisting behaviors caused by preoccupation (e.g., checking defects), and significant distress or impaired functioning. Presence of BDD is low, from 0.7 to 3.2% of the general population (Barahmand and Shahbazi, 2015; Schieber et al., 2015), although it is suspected that persons with this type of alteration do not seek help (a sort of silent disorder) or do so only in the severest of cases due to its social, family or personal consequences (Castle et al., 2004).

Thus the *dysmorphic concern* (DC) dimensional construct (Oosthuizen et al., 1998), refers to excessive preoccupation with a slight or imagined defect in physical appearance (Oosthuizen et al., 1998; Onden-Lim and Grisham, 2013). As given in the DSM-5 (American Psychiatric Association [APA], 2013, p. 247), has been proposed as a wider concept, beyond concern for physical appearance in general linked to normative body dissatisfaction (Rodin et al., 1984). It has been suggested that in addition to the slight or imagined defect, this construct should also be extended to the presence of social avoidance and behaviors directed at controlling the defect in appearance (Onden-Lim and Grisham, 2013). From this viewpoint, the DC construct would more closely approach the diagnostic definition of BDD, and perhaps one of more clinical usefulness.

In any case, the evaluation of the DC construct may be useful for identifying BDD proneness or risk. This is particularly important among young people, as it is known that the first symptoms of BDD start in adolescence at around 16 years of age (Phillips et al., 2005; Bjornsson et al., 2013). One study found that 9% of young people with a mean age of 12 could be at risk of developing BDD (Mastro et al., 2016). Data suggest that the presence of BDD in university students is from 2.3 to 5.8% (Bartsch, 2007; Taqui et al., 2008). When DC was measured in university students, the figure was over 19% of those evaluated (Barahmand and Shahbazi, 2015). However, although all cases of DC do not lead necessarily to a formal diagnosis of BDD, this does not mean exemption from distress, and it may define a situation of personal risk.

Some results have suggested that DC may not be an automatic process related to a personal standard, but rather a partially conscious bias toward stimuli related to appearance (Onden-Lim et al., 2012), conditioned by the need to fit into the social standard of body and appearance, and possibly mediated by sensitivity to rejection by others (Lavell et al., 2014b). It is possible that social anxiety, experiences of victimization and other variables like perfectionism, contribute to developing and maintaining these DC (Bartsch, 2007; Anson et al., 2012; Lavell et al., 2014a,b; Webb et al., 2015; Mastro et al., 2016). Since BDD (and DC) are located in the obsessive–compulsive spectrum, it is expectable for common cognitive processes, such as rumination or high self-consciousness, to also contribute to their genesis and maintenance, as in other disorders in this group (Onden-Lim et al., 2012; Onden-Lim and Grisham, 2013; Lavell et al., 2014a). This also occurs in other more specific and emotional symptoms, basically anxiety and depression (Phillips, 2009; Rodríguez-Testal, 2013; Keating et al., 2016).

All mentioned above suggests the need for clinically useful tools for knowing and identifying the processes related to DC (and BDD). Therefore, the following goals are posed: (1) Adapt the factor structure of the *Body Dysmorphic Disorder Examination Self-Report* (BDDE-SR; Reiter, 1996) and the *Dysmorphic Concern Questionnaire* (DCQ; Oosthuizen et al., 1998) to Spanish. Both instruments include the DC construct. The DCQ is more general or strictly related to the concept as cognition, while the BDDE-SR has a broader definition which includes preoccupation and negative evaluation of appearance, excessive importance given to appearance in self-evaluation, avoidance of places and activities, body camouflaging, and body checking, (2) Find the psychometric reliability (internal consistency and retest) and validity (content, convergent, and discriminant) properties of the BDDE-SR and DCQ tests, and (3) Analyze the risk of BDD by detecting DC using the BDDE-SR and DCQ.

## Materials and Methods

### Participants

The sample consisted of 966 participants (34.12% university students and 65.88% non-university general population), 62.7% women with an average age of 32.44 (*SD* = 13.33; range 18–65 years). The average social class index (SCI) according to Hollingshead (1975) was 48.72 (*SD* = 21.81) (mean social class).

A total of 46 participants were excluded because they currently had some psychological disorder, leaving a final sample of 920 subjects. The student group was recruited by incidental sampling. The participants from the general population were recruited by snowballing, so the characteristics of these two groups would be close to the general population. Many students put us in contact with relatives and acquaintances (not university students) who, in turn, contacted other friends and acquaintances (not university students). All the participants received an information sheet explaining the general characteristics of the evaluation and signed their consent for participating. The Clinical Research Ethics Committee of Cádiz (University Hospital Puerta del Mar) approved the research. The principles of the World Medical Association (Declaration of Helsinki) were followed.

### Instruments

#### Demographic and Current Symptom Questionnaire (Tool Developed by Authors)

This self-evaluation identified the SCI (Hollingshead, 1975), current illnesses, psychopathological antecedents, history and duration of symptoms, psychopharmacological treatments, and use of other drugs.

#### Body Dysmorphic Disorder Examination Self-Report (Reiter, 1996)

This 26-item test was designed to evaluate dysmorphic alterations of BI. Items are scored from 0 to 6, except for Item 16 which is dichotomous (YES/NO). Items 9, 10, 11, and 16 have two parts. The final score is the sum of all the items. A high score shows characteristic indicators of BDD (overvalued ideas and avoidance and checking behaviors associated with negative BI). The psychometric properties of the original version are adequate: test–retest reliability (*r* = 0.89), internal consistency (α = 0.94), and convergent validity with other measures of BI disorders (*r* = 0.69–0.83) (Reiter, 1996; Rosen and Ramirez, 1998).

#### Goldberg General Health Questionnaire (GHQ-28) Spanish Version by Lobo et al. (1986)

This is a screening test that gives an overall evaluation of health and social dysfunction. It consists of 28 items grouped in four subscales on somatic symptoms, anxiety, social dysfunction, and severe depression. In this study, the anxiety (GHQ-A), somatic symptoms (GHQ-S), and depression (GHQ-D) subscales were used. It has adequate reliability (test–retest, *r* = 0.90) and validity (sensitivity from 44 to 100% and specificity from 74 to 93%). In this study, internal consistency for GHQ-A was α = 0.80, GHQ-D was α = 0.70 and GHQ-S was α = 0.75.

#### Dysmorphic Concern Questionnaire (DCQ; Oosthuizen et al., 1998)

This questionnaire is comprised of seven items with the characteristic concerns of BDD, for example: …*Been very concerned about some aspect of your physical appearance?* It has a Likert-type response format (four choices, from 0 to 3 points). High scores show more preoccupation with physical appearance. The designers of the instrument found internal consistency of α = 0.80–0.88 and strong correlation with the BDDE (Pavan et al., 2008). Preliminary results for the overall DCQ score in Spanish university students were: α = 0.82 and retest *r* = 0.78 (Valdés-Díaz et al., 2013), and correlations were found with measures of alteration of BI (IMAGEN Questionnaire *r* = 0.61).

#### The Ruminative Response Scale (RRS; Nolen-Hoeksema and Morrow, 1991), Spanish Version by Hervás (2008)

This evaluates the presence of a ruminating response pattern. It consists of 22 items distributed in two factors: rumination and reflection. The first represents the negative component of the ruminating style and the second a more adapted component. The Spanish version has shown good retest reliability and internal consistency indices (rumination α = 0.80, reflection α = 0.74), and adequate factor, convergent and incremental validity. In this study only the rumination factor was applied (REP) with α = 0.77.

#### The Cuestionario Imagen [Image Questionnaire] (Solano and Cano, 2010)

This evaluates dissatisfaction with BI. It is comprised of 38 items (in Likert-type format 0–4 points) grouped in three factors: cognitive-emotional dissatisfaction (ICOG), perceptive dissatisfaction (IPER), and behavioral dissatisfaction (IBEH). It has satisfactory psychometric indices: α = 0.91, retest stability = 0.97 for the total score and validity (construct and convergent: with the BSQ and the CIMEC) (Solano and Cano, 2010). In this study, internal consistency was α = 0.95 for ICOG; α = 0.87 for IPER; α = 0.72 for IBEH.

#### The Revised Self-consciousness Scale (Scheier and Carver, 1985), Spanish Version by Banos et al. (1990)

This is made up of 22 items which evaluate the tendency of individuals to direct their attention outside or within themselves. It has three factors: (1) Private self-consciousness (PRISC), (2) Public self-consciousness (PUBSC), and (3) social anxiety, which was not used in this study. It has adequate reliability (α PRISC = 0.75, α PUBSC = 0.92), and validity (content and construct). In this study, consistency was α = 0.75 for PRISC, and α = 0.77 for PUBSC.

### Procedure

The translation and adaptation of the BDDE-SR and DCQ scales to Spanish were done following the recommendations of Muñiz et al. (2013) using the back-translation method with two translators, one of them familiar with the Spanish culture and the other familiar with the United States. The first translator translated the scales into Spanish and then this translation was translated back into English again. These versions were compared with the original English versions for accuracy.

### Data Analysis

The sample was divided at random into two halves for cross validation of the instruments. Two Exploratory Factor Analyses (EFA), one for the BDDE and another one for the DCQ, were done with Sample 1 (*n* = 460), on the polychoric correlations matrix with Robust Diagonally Weighted Least Squares (RDWLS) and Direct Oblimin rotation. Three models were tested by Confirmatory Factor Analysis (CFA) with Sample 2 (*n* = 460), each for the BDDE and DCQ, with the RDWLS method using the asymptotic covariance matrix. Chi squared, Comparative Fit Index (CFI), Non-Normed Fit Index (NNFI), Goodness of Fit Index (GFI), Adjusted Goodness of Fit Index (AGFI), which must be >0.90 (Baumgartner and Homburg, 1996) were used to test the overall fit of models. In addition to these indices, the Root Mean Square Error of Approximation (RMSEA) and its confidence interval at 90%, which must be ≤0.05 for a good fit, and between 0.05 and 0.08 for an acceptable fit. The Standardized Root Mean Square Residual (SRMR), which must be ≤0.05 for a good fit, and between 0.05 and 0.10 for an acceptable fit (Schermelleh-Engel and Moosbrugger, 2003), were also calculated.

For descriptive purposes, mean gender and age were compared (*n* = 920) with the BDDE-SR-28 scores (name after elimination of items in CFA) and DCQ, and the quartiles were found for both tests to determine BDD risk.

Cronbach’s α and test–retest reliability indices were found. To study the validity of BDDE-SR-28 test content, a jury of experts comprised of 15 clinical psychologists evaluated item adequacy for their respective constructs on a Likert type scale (0–5 points). The Aiken *V* was found from the average scores of each judge. The criterion of reference for item adequacy was *V* > 0.70 (Charter, 2003). For convergent validity, bivariate Pearson’s Correlation analyses were conducted. For discriminant validity, a one-way analysis of variance (ANOVA) was done of this variable (subjects at dysmorphic risk or not) with participants in the ≥75th percentile on the BDDE-SR-28 and the DCQ (subjects at dysmorphic risk) over the factors in the IMAGEN test, the factors on the GHQ-28 test, rumination (RRS) and PRISC and PUBSC (Self-Consciousness Scale). Statistical analyses were done with the SPSS, Lisrel 8.7 (for CFA), and Factor 10.4.01 programs (for EFA; Lorenzo-Seva and Ferrando, 2006).

## Results

### Preliminary Analysis

The sample was divided into two halves (two groups). The sociodemographic variables (gender, age, and SCI, *p* > 0.05) and DCQ and BDDE-SR overall measurements (*p* > 0.05), were equivalent in both groups before the psychometric analysis (**Table 1**^1^).

**Table 1:** Comparison of means between Sample 1 and Sample 2 of sociodemographic variables and total scores on DCQ and BDDE-SR.

	Sample 1 (*n* = 460)*M* (*SD*), *n* (%)	Sample 2 (*n* = 460)*M* (*SD*), *n* (%)	*t*/χ^2^ (*df*)	*p*
Age	32.52 (13.11)	32.37 (13.15)	0.17 (918)	0.866
SCI	50.11 (22.64)	47.40 (21)	0.86 (918)	0.391
BDDE-SR (range 0–146)	32.81 (27.44)	31.08 (25.42)	0.99 (918)	0.321
DCQ (range 0–28)	3.92 (3.63)	3.97 (3.54)	-0.23 (918)	0.818
Gender	Men: 170 (18.5%)Women: 290 (31.5%)	Men: 173 (18.8%)Women: 287 (31.2%)	0.042 (1)	0.838
Percentile 75 BDDE-SR(46 points)	24.35%	25.43%		
Percentile 75DCQ (6 points)	24.35%	27.82%		

### Exploratory and Confirmatory Factor Analyses of the BDDE-SR Scale

An EFA was done of the BDDE-SR test with Sample 1 (*n* = 460), which showed adequate values in the KMO (0.92, 95% CI = 0.920–0.921) and Bartlett’s sphericity [χ^2^(435) = 7179.9, *p* < 0.001] tests. However, Items 1, 10a, 12, and 15 showed communalities below 0.40. Parallel analysis recommended a two-factor solution. The first factor (related with dissatisfaction/preoccupation with BI) included Items 1, 2, 3, 4, 5, 6, 7, 8, 9a, 9b, 10a, 10b, 11a, 11b, 12, 13, 14, 15, 22, 23, and 26 and the second factor (related with BI avoidance behavior) included Items 16a, 16b, 17, 18, 19, 20, 21, 24, and 25 (the content of these items may be found in the Supplementary Material, Table 1). All items were well represented in the factor they were intended to measure except Items 22 and 23, related to BI avoidance behavior. These two factors explained 55% of the variance.

**FIGURE 1:**
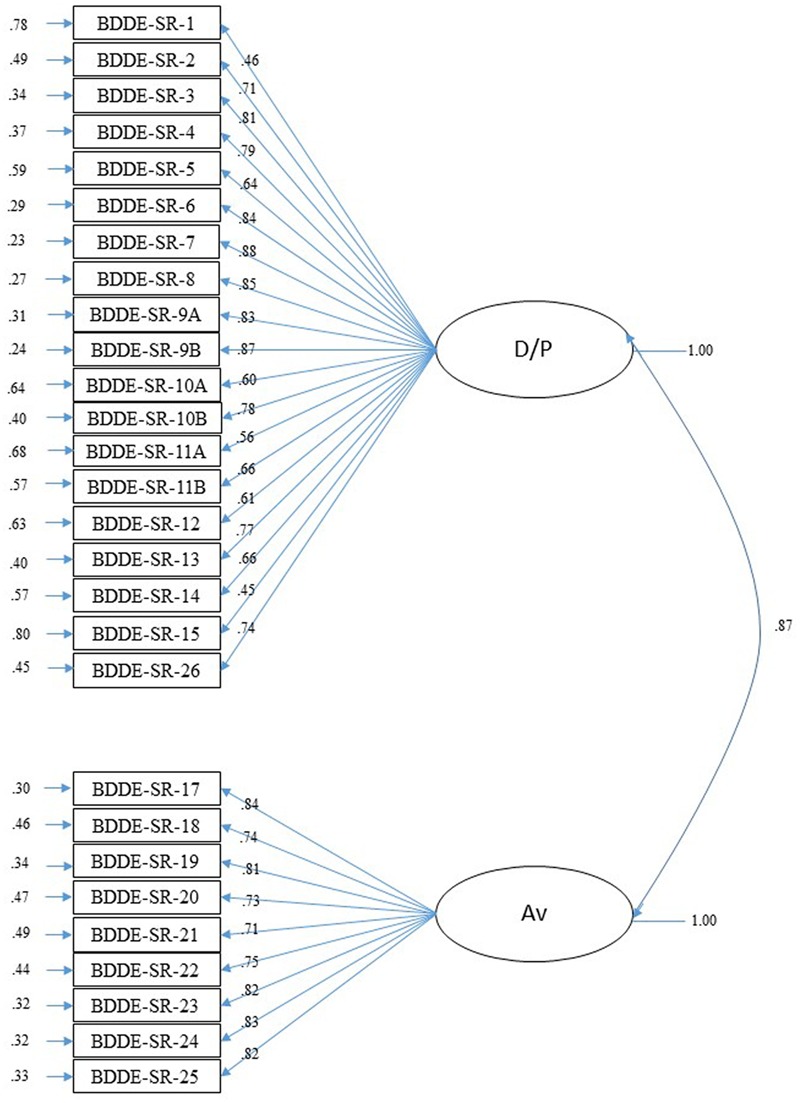
Confirmatory Factor Analysis for BDDE-SR-28. Completely standardized solution. D/P, dissatisfaction/preoccupation with body image; AV, body image avoidance behaviors.

The CFA was done with Sample 2 (*n* = 460). A first-order factor structure was tested with the data from the EFA. Fit indices found were adequate (Model 1, **Table 2**), although the indices of modification suggested some changes that were considered under the theoretical cover of the model and the approval of the expert judges (transfer of Items 22 and 23 to Factor 2 and elimination of Items 16a and 16b because they showed low factor loading, 0.05 and 0.29, respectively). The new model analyzed (Model 2, **Table 2**) with the modifications described had better fit indices than Model 1, although the correlation between factors in both Models 1 and 2 was very high *r* = 0.87. Given the high correlation, it was decided to try a one-dimensional model as suggested by the scale’s designer (Model 3, **Table 2**). RMSEA was inadequate and the chi-square found was higher than for Models 1 and 2. The fit indices showed that the model 2 was the best (see **Table 2** and **Figure 1**). Hereinafter this reduced version of the scale is called the BDDE-SR-28.

**Table 2:** Fit indices of models analyzed with Confirmatory Factor Analysis: BDDE-SR.

Model	χ^2^	*df*	*p*	GFI	NFI	CFI	AGFI	SRMR	RMSEA (90% CI)
Model 1: two-factor model found by EFA (30 items)	1430.54	404	<0.001	0.99	0.97	0.98	0.98	0.073	0.074 (0.070–0.083)
Model 2: two-factor model with modifications (28 items)	1300	**349**	**<0.001**	**0.99**	**0.97**	**0.98**	**0.99**	**0.064**	**0.077 (0.073–0.082)**
Model 3: one-dimensional model (28 items)	1663.63	350	<0.001	0.99	0.96	0.97	0.98	0.071	0.091 (0.086–0.095)

*^∗^The values in bold indicate that it is the best model.*

### Exploratory and Confirmatory Factor Analyses of the DCQ Scale

**FIGURE 2:**
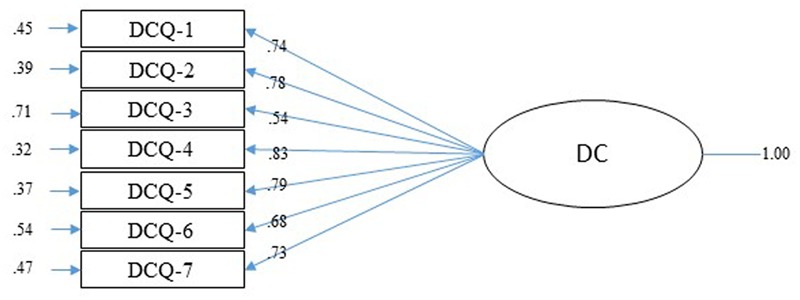
Confirmatory Factor Analysis of DCQ. Completely standardized solution.

The EFA of the DCQ showed adequate values for the KMO (0.85, 95% CI = 0.83–0.88) and Bartlett’s sphericity [χ^2^ (21) = 1231.8, *p* < 0.001] tests. Parallel analysis recommended a one-factor solution with an eigenvalue of 4.21 (explaining 60% of the variance). The fit indices found after CFA were all adequate: χ^2^(13) = 58.79, *p* < 0.001; NFI = 0.98, CFI = 0.98, GFI = 0.99, AGFI = 0.98, SRMR = 0.058 and RMSEA = 0.083 (CI = 0.062–0.11). The factor loading varied from 0.54 to 0.83 (see **Figure 2**).

### Descriptive Analysis

Statistically significant differences were found between the genders in total scores on the BDDE-SR-28 and DCQ scales, where women had the highest scores on both tests [BDDE-SR-28: *t*(787.52) = -6.62, *p* < 0.001; DCQ: *t*(774.31) = -4.19, *p* < 0.001]. The sample was divided into two groups by age (<27 and ≥27 years), taking the median in this variable as reference, and the means over the BDDE-SR-28 and DCQ variables were compared. The youngest had a higher mean on the BDDE-SR-28: *t*(882.99) = 5.47, *p* < 0.001 and DCQ: *t*(866.93) = 6.81, *p* < 0.001. The score quartiles were calculated for both tests to be able to find risk scores and subjects, finding 25% of the participants in the third quartile (≥75th percentile) on the BDDE-SR-28 and 26.09% on the DCQ. However, the subjects in the ≥75th percentile on both tests represented 14.71% of the sample (*n* = 136). These subjects were considered to be at dysmorphic risk.

### Psychometric Properties: Reliability and Validity

A jury of experts (*n* = 15 clinical psychologists, average clinical experience = 16.36 years, *SD* = 10.26) evaluated the validity of the BDDE-SR-28 scale content after eliminating the Items 16a and 16b, and Items 22 and 23 were transferred to the avoidance behaviors factor. All the items had valid coefficients (Aiken *V*) of *V* = 0.77 (Item 14) to *V* = 0.98 (Item 17).

The psychometric properties were found with the complete sample (*n* = 920). Internal consistency for the total BDDE-SR-28 eliminating the items 16a and 16b was α = 0.94. The Cronbach’s α for the BI preoccupation/dissatisfaction factor (19 items) was α = 0.93 and for the BI avoidance factor (nine items) was α = 0.87. Retest reliability = 0.96 (average interval of 1 month). Internal consistency for the DCQ was α = 0.85 and retest reliability = 0.87 (average interval of 1 month).

To determine convergent validity, a Pearson’s correlation analysis was done of the IMAGEN subscales (ICOG, IPER, IBEH), the BDDE-SR-28 dissatisfaction/preoccupation and avoidance behaviors factors and the total DCQ scale score. **Table 3** shows the positive statistically significant correlations of all the measures. Particularly strong is the relationship between DCQ and the BDDE-SR-28 dissatisfaction/preoccupation factor, and between this one and the IMAGEN test ICOG factor.

**Table 3:** Pearson’s correlation matrix of DCQ, BDDE-SR-28, and IMAGEN domain scores.

Variables	1	2	3	4	5	6
1. D/P BDDE-SR-28	-					
2. AV BDDE-SR-28	0.739**	-				
3. DCQ	0.657**	0.557**	-			
4. ICOG	0.647**	0.603**	0.526**	-		
5. IPER	0.435**	0.484**	0.366**	0.648**	-	
6. IBEH	0.413**	0.420**	0.394**	0.487**	0.356**	-
*Mean*	25.36	7.67	3.94	25.87	3.30	0.70
*SD*	18.18	8.96	3.58	19.47	5.42	1.91

*1, dissatisfaction/preoccupation with body image (BDDE-SR-28); 2, body image avoidance behaviors (BDDE-SR-28); 3, total DCQ score; 4, ICOG cognitive-emotional dissatisfaction (IMAGEN); 5, IPER perceptive dissatisfaction (IMAGEN); 6, IBEH dissatisfaction behaviors (IMAGEN). All values are ^∗∗^*p* < 0.001.*

To analyze the discriminant validity of both measures, means of the subjects considered at high dysmorphic risk (*n* = 136) vs not at risk (*n* = 784) and the IMAGEN, GHQ-28, REP, and self-consciousness factors were compared. The results showed statistically significant differences in all the comparisons with high-risk subjects scoring highest on the factors in the IMAGEN: ICOG *t*(918) = -16.01, *p* < 0.001; IPER *t*(918) = -12.48; *p* < 0.001; IBEH *t*(918) = -10.44, *p* < 0.001 and on the rest of the measures: GHQ-A: *t*(918) = -9.02, *p* < 0.001; GHQ-S: *t*(918) = -8.60, *p* < 0.001; GHQ-D: *t*(918) = -7.45, *p* < 0.001, REP (RRS): *t*(918) = -11.02, *p* < 0.001; PUBSC: *t*(918) = -8.80, *p* < 0.001; PRISC: *t*(918) = -7.98, *p* < 0.001.

## Discussion

The purpose of this study was to adapt the DCQ and BDDE-SR scales to Spanish and study their factor structures, to find their psychometric properties and analyze BDD risk by detecting DC with these tests.

The results of EFA and CFA corroborated the one-dimensional structure of the DCQ for which adequate goodness-of-fit indices were found. For the BDDE-SR, both analyses determined the existence of two different but very closely related factors. A one-dimensional model was tested, however, the fit indices were not entirely adequate. In spite of this, the one-dimensional option may not be discarded. A one dimensional model is more restricted than a model with more factors, and therefore, it is to be expected that such a wide scale have a less parsimonious fit, unlike the DCQ which fits one-dimensionally very well because it is a very short scale. However, in view of its better goodness-of-fit indices and the suggestions of the experts (keeping in mind the theoretical corpus), we decided on the model with two first-order factors. A factor more related to the cognitive-emotional component of BI (preoccupation and dissatisfaction) and another more related to BI avoidance behaviors were found. Both components were close to the BI dimensions suggested by Cash and Smolak (2011). One was an evaluative or comparative component concentrating on pleasing/displeasing and satisfaction/dissatisfaction with physical appearance, and the other a component related to dedication or investment in appearance, in this case, more clearly behavioral: care, attention and body-related behavior.

CFA of the BDDE-SR recommended elimination of two items because of their low factor loading (16a and 16b). These items are the only ones that have a different response format (YES/NO answer choice) and it could be problematic to consider them with the rest of the items which have scores ranging from 0 to 6 points. After these results and evaluation by experts of the adequacy of the items with their respective constructs, we validated a reduced Spanish version of the BDDE-SR with adequate goodness-of-fit indices and psychometric properties, the BDDE-SR-28.

On the other hand, even though Reiter (1996) was based on a one-dimensional model, the 10 items he considered for the diagnosis of BDD would be represented in six items pertaining to the factor identified as dissatisfaction/preoccupation, and four pertaining to the factor named avoidance. Consequently, neither the consideration of the two factors nor the elimination of two items affected to the original test.

Adequate reliability indicators were found for both the DCQ and the BDDE-SR-28 in a general population sample not formed exclusively of university students. Internal consistency was satisfactory for both scales and the retest stability showed good characteristics over an average time interval of 1 month. Validity indicators were adequate. Both instruments showed similar results and in the direction predicted for convergent validity, especially the relationship between the BDDE-SR-28 and DCQ dissatisfaction/preoccupation factor and the IMAGEN test cognitive-emotional dissatisfaction factor. Because of the DCQ’s adequate psychometric properties and high correlations with the IMAGEN and BDDE-SR-28 factors, we can recommend its application as a screening scale for evaluating DC.

In discriminant validity, the highest scores (75th percentile in DCQ and total BDDE-SR-21) were significantly related to high BI dissatisfaction (IMAGEN test), greater presence of emotional symptoms (GHQ-A, GHQ-D, and GHQ-S), rumination (REP), and self-consciousness (PRISC and PUBSC) (Phillips, 2009; Onden-Lim et al., 2012; Onden-Lim and Grisham, 2013; Rodríguez-Testal, 2013; Lavell et al., 2014a). Therefore, the adapted measures may be considered useful for specifically cognitive evaluation of DC (DCQ), as well as broader and behavioral (BDDE-SR-28).

With the adaptation of these tests, it was also intended to back the “dysmorphic concern” dimensional construct beyond BI dissatisfaction, in which preoccupation for one or more perceived defects and a series of associated behaviors are present on a continuum where BDD would be at the far end (Oosthuizen et al., 1998; Onden-Lim and Grisham, 2013). In view of the results, we consider both the BDDE-SR-28 and the DCQ adequate for detecting DC in the general population, and therefore, risk of developing BDD. The quartiles on both tests show that a significant part of the sample (from 25 to 26%) had DC, higher than what has been found in other studies with participants under 20 years of age (Barahmand and Shahbazi, 2015) and close to other studies in which only university students participated (Bartsch, 2007). Keeping in mind that in our sample the age range was wider, this percentage of persons with DC must be considered high. Therefore, risk of BDD should not be set exclusively at the 75th percentile, but with coincidence in this percentile in both tests, since this is already clearly showing suffering and distress. Following this criterion, 14.71% of the sample had significant scores on both tests, much closer to the results that include university students, although with gender differences for both scales, not usually found for BDD (Phillipou and Castle, 2015), although there are studies that show a higher prevalence in women (Buhlmann et al., 2010; Schieber et al., 2015). Results for differences in age coincide with those in the literature (for a review, see Veale et al., 2016), in which the youngest are at the greatest risk of developing BDD.

This study had some limitations that should be born in mind. It was a cross-sectional design with no follow-up of subjects found with DC. It would have been interesting to have followed up on them to find out how many of those subjects at risk actually develop BDD. It would also be very important to establish the level of distress or suffering of individuals with DC, regardless of whether a BDD diagnosis is made at some time, so the sense of the construct can be analyzed in depth and described in detail. Apart from this, and to analyze the validity of criterion, a group of subjects diagnosed with BDD could have been included to find out if the DCQ and especially the BDDE-SR-28 tests were able to discriminate between the clinical and non-clinical populations and the clinical population and one identified as “dysmorphic risk.” Reiter (1996) demonstrated that the BDDE-SR scale was able to predict the clinical state of subjects diagnosed with BDD. Other researchers are encouraged to consider this possibility with the reduced version adapted to Spanish which this study has contributed. Thus in future studies, the predictive ability of these instruments and the DC construct will be tested for diagnosis of BDD as well as detailed description of the functional and emotional characteristics of DC.

In spite of its limitations, this study does have some strengths. In Spain, many adapted instruments are available for evaluating negative BI, but they are closer to detection of eating disorders. There are hardly any self-reported measures validated with Spanish samples for detection of BDD, even though it is a more prevalent disorder than anorexia or bulimia nervosa (Hoek, 2006). Most individuals with BDD do not seek psychological treatment for their problem (Castle et al., 2004), and it is more common for them to seek medical treatment (e.g., plastic surgery, dermatological or dental treatments), which far from improving their problem, contribute to its becoming chronic (Sarwer and Crerand, 2008; Veale et al., 2016). The BDDE-SR has been employed as a diagnostic instrument for BDD in individuals who request cosmetic surgery (Sarwer et al., 1998) and the DCQ for dermatological treatment (Stangier et al., 2003). Having validated scales which detect DC will enable healthcare professionals in the Spanish-speaking world to make a more accurate diagnosis and prescribe a treatment more suitable to the patient’s needs.

## Author Contributions

CS-C, SP-G, and JR-T contributed to conception and design of research, analysis and interpretation of data, drafting and revising manuscript. MV-D, MB-H, and MN-G contributed to the data collection, setting up the data base, writing and revising the work critically. RM-C has been the statistical advisor. All authors read and approved the final manuscript.

## Conflict of Interest Statement

The authors declare that the research was conducted in the absence of any commercial or financial relationships that could be construed as a potential conflict of interest.

## Footnotes

1 The descriptive analyses may be found by subsamples “student vs general population” in the Supplementary Material, Table 2.
