# Supplementary material for: Validation of Spanish Language Evaluation Instruments for Body Dysmorphic Disorder and the Dysmorphic Concern Construct
Source: Front Psychol. 2017 Jun 30;8:1107. doi: 10.3389/fpsyg.2017.01107 (PMC5492913; doi:10.3389/fpsyg.2017.01107)
Supplement: Supplementary file 1 [file Data_Sheet_1.docx]

Supplementary material

Table 1.

Standardized Factor Loading and Communalities of the 30-Item of BDDE-SR two Factor Model Derived From Exploratory Factor Analysis (EFA)

| Items | Factor 1^*^ | Factor 2^**^ | Communalities |
| --- | --- | --- | --- |
| 6. Over the past four weeks: How often have you thought about your appearance feature AND felt upset as a result? | .923 |  | .796 |
| 3. Over the past four weeks: How dissatisfied have you been with your appearance feature? | .906 |  | .702 |
| 2. Over the past four weeks: How frequently have you checked-out your appearance feature (for example, looked at it, felt it, measured it in some way) in order to evaluate the extent of the problem? | .815 |  | .590 |
| 7. Over the past four weeks: How much have you worried or felt embarrassed about your appearance feature when you were in public areas such as shopping malls, grocery stores, city streets, restaurants, movies, clubs, buses or planes, waiting in lines, parks or beaches, public restrooms, or other areas where maínly there were people you didn't know? (When answering, think about how many of these situations you worry in and how intense· your worrying is.) | .804 |  | .703 |
| 9b. Over the past four weeks, how upset have you become when you felt someone was noticing or paying attention to your appearance feature? (When answering, think about whether you felt differently depending on who the person was that noticed.) | .773 |  | .659 |
| 4. Over the past four weeks, how dissatisfied have you been with your overall appearance? | .754 |  | .555 |
| 26. Over the past four weeks, how often have you compared your appearance with the appearance of other people around you or in magazines or television? Include both positive and negative comparisons | .736 |  | .559 |
| 8. Over the past four weeks: How much have you worried or felt embarrassed about your appearance feature when you were in social settings with co-workers, acquaintances, friends, or family members (for example, at work, parties, family gatherings, meetings, talking in groups, having a conversation, dating or going on an outing with others, speaking to a boss or supervisor)? | .717 |  | .633 |
| 9a. Over the past four weeks, how often have you felt that other people were noticing or paying attention to your appearance feature? (Include times when you realize you might be imagining it.) | .715 |  | .605 |
| 10b. Over the past four weeks, how upset have you become when someone commented — positively or negatively — on your appearance feature? (When answering, think about whether you felt differently depending on who the person was that made the comment.) | .691 |  | .551 |
| 12. Over the past four weeks, how important has appearance been in how you evaluate yourself as a person? Before answering, think about other things that influence how you judge yourself, such as personality, intelligence, work or school performance, quality of your relationships with others, ability in other areas, and so on. Compared to these (and maybe others), how much importance have you given to appearance when evaluating yourseIf? | .619 |  | .332 |
| 22. Over the past four weeks, how much have you deliberately dressed, made yourself up, or groomed yourself in some special way in order to cover up your appearance feature or distract attention from it? This can include avoiding certain clothes or cosmetics. (This is called “camouflaging.”) | .582 |  | .497 |
| 13. Over the past four weeks, how negatively (if at all) have you thought of yourself as a person as a result of your appearance feature? This question is not asking whether you think your appearance is attractive or unattractive. Rather, it is asking how much your appearance made you feel that you had a personal flaw or were undesirable or inadequate in a non-physical way | .576 |  | .552 |
| 23. Over the past four weeks, how frequently have you deliberately changed your posture or body movements (such as the way you stand or sit, where you put your hands, how you walk, what side of yourself you show to other people, etc.) in order to hide your appearance feature or distract people’s attention from it? | .574 |  | .551 |
| 11a. Over the past four weeks, how often did someone do something to you or for you that you think was a result of your appearance feature? | .532 |  | .401 |
| 1. Over the past four weeks: How common have you felt it is far people your age and sex to have an appearance feature just like the one you believe you have? | .530 |  | .247 |
| 11b. Over the past four weeks, how upset did you become when someone did something to you or for you because of your appearance feature? (When answering, think about whether you felt differently depending on who the person was.) | .503 |  | .434 |
| 14. Over the past four weeks, how negatively (if at all) have you felt other people evaluated you as a person as a result of your appearance feature? Again, this question is not asking how attractive or unattractive other people thought you were. Rather, it is asking how much you thought your appearance made other people see you as undesirable or inadequate in some non-physical way. | .457 | .363 | .504 |
| 5. Over the past four weeks: How frequently have you tried to get reassurance from someone that your appearance feature isn't as bad or abnormal as you think it is? | .396 | .347 | .413 |
| 10a. Over the past four weeks, how often did someone unexpectedly make a positive or negative comment on your appearance feature? (Only include comments that came “out of the blue,” not comments you might have tried to get from the person.) | .391 |  | .294 |
| 15. Over the past four weeks, how attractive physically did you feel other people thought you were? (If friends view you differently than strangers, how attractive on average did you feel people thought you were?) | .327 |  | .193 |
| 18. Over the past four weeks, how much have you avoided work or other social situations with friends, relatives, or acquaintances because you felt uncomfortable about your appearance feature? Social situations could include going to work or school, parties, family gatherings, meetings, talking in groups, having a conversation, hanging out with others at work, dating or going on an outing with others, or speaking to a boss or supervisor |  | .827 | .768 |
| 16a. Over the past four weeks, have you ever thought your appearance feature might not be as bad as you generally think, or have there been times that you’ve felt significantly better about your appearance feature? |  | .806 | .491 |
| 16b. Over the past four weeks, have you ever felt that your appearance was basically normal? |  | .799 | .582 |
| 19. Over the past four weeks, how much have you avoided close physical contact with others because of your appearance feature? This includes sexual activity as well as other close contact such as shaking hands, hugging, kissing, or dancing close. |  | .699 | .652 |
| 21. Over the past four weeks, how much have you avoided physical activities such as exercise or outdoor recreation because of feeling self-conscious or uncomfortable due to your appearance feature? |  | .687 | .645 |
| 17. Over the past four weeks, how much have you avoided public areas because you felt uncomfortable about your appearance feature? (Such areas might include shopping malls, grocery stores, city streets, restaurants, movies, clubs, buses or planes, waiting in lines, parks, beaches, public restrooms, or other areas where mainly there would be people you don’t know.) |  | .651 | .701 |
| 25. Over the past four weeks, how frequently have you avoided other people seeing your body unclothed because you felt uncomfortable about your appearance feature? This includes not letting your spouse, partner, roommate, etc., see you without clothes, or people in public settings, such as in health club showers or changing rooms | .377 | .469 | .536 |
| 24. Over the past four weeks, how often have you avoided looking at your body, particularly at your appearance feature, in order to control feelings about your appearance? This includes avoiding looking at yourself clothed or unclothed, either directly or in mirrors or windows. | .371 | .451 | .506 |
| 20. Over the past four weeks, when making contact physically with others (for example, lovemaking, hugging, shaking hands, kissing, dancing close), how often have you tried to restrict the amount of actual contact that occurs (for example, by changing your posture, limiting your movement, or  preventing contact with certain body parts)? | .394 | .426 | .503 |

^*^ *Factor 1: Dissatisfaction /preoccupation with body image*

^**^ *Factor 2: Body image avoidance behavior*

Table 2. Comparison of means between Student Sample and general population Sample of sociodemographic variables and total scores on DCQ and BDDE-SR

|  | Student Sample (*n*=314)  *M* (*SD*), *n* (%) | General Population Sample (*n*=606)  *M* (*SD*), *n* (%) | *t/ χ*^2^ (*df*) | *p* |
| --- | --- | --- | --- | --- |
| Age | 20.38 (1.31) | 38.72 (12.41) | 35.92 (918) | .000 |
| SCI | 51.23 (20.35) | 44.54 (23.61) | 2.07 (918) | .039 |
| BDDE-SR (range 0-146) | 35.01 (26.31) | 30.33 (26.40) | 2.57 (918) | .010 |
| DCQ  (range 0-28) | 4.23 (3.34) | 3.79 (3.70) | 1.75 (918) | .080 |
| Gender | Men: 111 (35.4%)  Women: 203 (65.6%) | Men: 232 (37.3%)  Women: 374 (62.7%) | .761 (1) | .383 |
| Percentile 75 BDDE-SR (46 points)  Percentile 75 DCQ  (6 points) | 24.52%  22.61% | 22.11%  21.29% |  |  |

Output Exploratory Factor Analysis

Method to handle missing values : Hot-Deck Multiple Imputation in Exploratory Factor Analysis (Lorenzo-Seva & Van Ginkel, 2016)

Missing code value : 999

Number of participants : 460

Number of variables : 30

Variables included in the analysis : ALL

Variables excluded in the analysis : NONE

Number of factors : 2

Number of second order factors : 0

Procedure for determining the number of dimensions : Optimal implementation of Parallel Analysis (PA) (Timmerman, & Lorenzo-Seva, 2011)

Dispersion matrix : Polychoric Correlations (Bayes modal estimation; Choi, Kim, Chen, & Dannels 2011)

Robust analyses : Bias-corrected and accelerated (BCa; Lambert, Wildt & Durand, 1991)

Number of bootstrap samples : 500

Asymptotic Covariance/Variance matrix : estimated using bootstrap sampling

Bootstrap confidence intervals : 95%

Method for factor extraction : Robust Diagonally Weighted Least Squares (RDWLS)

Correction for robust Chi square : Robust Mean-scaled

Rotation to achieve factor simplicity : Direct Oblimin

Value of parameter gamma : 0.0000

Clever rotation start : Weighted Varimax

Number of random starts : 10

Maximum mumber of iterations : 100

Convergence value : 0.00001000

--------------------------------------------------------------------------------

UNIVARIATE DESCRIPTIVES

Variable Mean Confidence Interval Variance Skewness Kurtosis

(95%) (Zero centered)

V 1 2.564 ( 2.38 2.75) 2.377 0.181 -0.322

V 2 1.924 ( 1.69 2.16) 3.796 0.841 -0.493

V 3 2.401 ( 2.21 2.60) 2.650 0.085 -0.827

V 4 1.786 ( 1.61 1.97) 2.255 0.522 -0.435

V 5 0.560 ( 0.44 0.68) 1.079 2.138 4.593

V 6 1.309 ( 1.11 1.50) 2.671 1.365 1.114

V 7 1.403 ( 1.21 1.60) 2.624 0.919 -0.236

V 8 1.392 ( 1.20 1.58) 2.583 1.016 0.131

V 9 1.083 ( 0.92 1.25) 1.906 1.589 2.296

V 10 1.207 ( 1.03 1.39) 2.269 1.424 1.451

V 11 0.956 ( 0.82 1.09) 1.240 1.737 3.901

V 12 1.107 ( 0.94 1.28) 2.004 1.515 1.951

V 13 0.283 ( 0.19 0.37) 0.582 3.838 18.240

V 14 0.355 ( 0.25 0.46) 0.787 3.522 14.875

V 15 1.963 ( 1.77 2.15) 2.498 0.287 -0.928

V 16 1.033 ( 0.87 1.20) 1.936 1.381 1.412

V 17 0.695 ( 0.56 0.83) 1.232 1.908 3.762

V 18 1.187 ( 1.02 1.36) 2.008 1.102 0.548

V 19 0.325 ( 0.22 0.42) 0.694 4.247 21.610

V 20 0.194 ( 0.13 0.26) 0.331 4.863 33.154

V 21 0.481 ( 0.35 0.61) 1.139 2.690 7.238

V 22 0.351 ( 0.24 0.46) 0.864 3.361 12.386

V 23 0.623 ( 0.48 0.77) 1.507 2.263 4.937

V 24 0.704 ( 0.55 0.86) 1.633 2.036 3.706

V 25 0.575 ( 0.43 0.72) 1.386 2.340 5.401

V 26 1.566 ( 1.33 1.80) 3.836 1.117 -0.000

V 27 1.248 ( 1.05 1.45) 2.862 1.393 0.933

V 28 0.739 ( 0.58 0.90) 1.779 2.109 4.022

V 29 1.111 ( 0.91 1.31) 2.704 1.462 1.144

V 30 1.758 ( 1.54 1.98) 3.386 0.872 -0.343

Polychoric correlation is advised when the univariate distributions of ordinal items are

asymmetric or with excess of kurtosis. If both indices are lower than one in absolute value, hen Pearson correlation is advised. You can read more about this subject in:

Muthén, B., & Kaplan D. (1985). A comparison of some methodologies for the factor analysis of non-normal Likert variables. British Journal of Mathematical and Statistical Psychology, 38, 171-189.

Muthén, B., & Kaplan D. (1992). A comparison of some methodologies for the factor analysis of non-normal Likert variables: A note on the size of the model. British Journal of Mathematical and Statistical Psychology, 45, 19-30.

BAR CHARTS FOR ORDINAL VARIABLES

Variable 1

Value Freq

|

0 62 | **************

1 19 | ****

2 174 | ****************************************

3 71 | ****************

4 89 | ********************

5 23 | *****

6 21 | ****

+-----------+---------+---------+-----------+

0 43.5 87.0 130.5 174.0

Variable 2

Value Freq

|

0 141 | ****************************************

1 104 | *****************************

2 77 | *********************

3 29 | ********

4 45 | ************

5 20 | *****

6 43 | ************

+-----------+---------+---------+-----------+

0 35.3 70.5 105.8 141.0

Variable 3

Value Freq

|

0 88 | ***********************

1 30 | ********

2 147 | ****************************************

3 49 | *************

4 106 | ****************************

5 27 | *******

6 12 | ***

+-----------+---------+---------+-----------+

0 36.8 73.5 110.3 147.0

Variable 4

Value Freq

|

0 126 | **********************************

1 68 | ******************

2 146 | ****************************************

3 37 | **********

4 68 | ******************

5 7 | *

6 7 | *

+-----------+---------+---------+-----------+

0 36.5 73.0 109.5 146.0

Variable 5

Value Freq

|

0 323 | ****************************************

1 60 | *******

2 51 | ******

3 9 | *

4 13 | *

5 2 |

6 1 |

+-----------+---------+---------+-----------+

0 80.8 161.5 242.3 323.0

Variable 6

Value Freq

|

0 201 | ****************************************

1 105 | ********************

2 68 | *************

3 29 | *****

4 26 | *****

5 11 | **

6 19 | ***

+-----------+---------+---------+-----------+

0 50.3 100.5 150.8 201.0

Variable 7

Value Freq

|

0 209 | ****************************************

1 56 | **********

2 99 | ******************

3 17 | ***

4 57 | **********

5 15 | **

6 6 | *

+-----------+---------+---------+-----------+

0 52.3 104.5 156.8 209.0

Variable 8

Value Freq

|

0 203 | ****************************************

1 65 | ************

2 100 | *******************

3 23 | ****

4 44 | ********

5 15 | **

6 9 | *

+-----------+---------+---------+-----------+

0 50.8 101.5 152.3 203.0

Variable 9

Value Freq

|

0 206 | ****************************************

1 129 | *************************

2 65 | ************

3 23 | ****

4 19 | ***

5 9 | *

6 8 | *

+-----------+---------+---------+-----------+

0 51.5 103.0 154.5 206.0

Variable 10

Value Freq

|

0 200 | ****************************************

1 122 | ************************

2 60 | ************

3 28 | *****

4 29 | *****

5 8 | *

6 12 | **

+-----------+---------+---------+-----------+

0 50.0 100.0 150.0 200.0

Variable 11

Value Freq

|

0 183 | ****************************************

1 181 | ***************************************

2 57 | ************

3 18 | ***

4 14 | ***

5 2 |

6 4 |

+-----------+---------+---------+-----------+

0 45.8 91.5 137.3 183.0

Variable 12

Value Freq

|

0 211 | ****************************************

1 116 | *********************

2 69 | *************

3 23 | ****

4 24 | ****

5 7 | *

6 9 | *

+-----------+---------+---------+-----------+

0 52.8 105.5 158.3 211.0

Variable 13

Value Freq

|

0 379 | ****************************************

1 50 | *****

2 21 | **

3 3 |

4 2 |

5 3 |

6 1 |

+-----------+---------+---------+-----------+

0 94.8 189.5 284.3 379.0

Variable 14

Value Freq

|

0 365 | ****************************************

1 57 | ******

2 21 | **

3 8 |

4 3 |

5 2 |

6 3 |

+-----------+---------+---------+-----------+

0 91.3 182.5 273.8 365.0

Variable 15

Value Freq

|

0 125 | **********************************

1 46 | ************

2 143 | ****************************************

3 33 | *********

4 95 | **************************

5 12 | ***

6 5 | *

+-----------+---------+---------+-----------+

0 35.8 71.5 107.3 143.0

Variable 16

Value Freq

|

0 247 | ****************************************

1 62 | **********

2 94 | ***************

3 16 | **

4 30 | ****

5 4 |

6 6 |

+-----------+---------+---------+-----------+

0 61.8 123.5 185.3 247.0

Variable 17

Value Freq

|

0 285 | ****************************************

1 83 | ***********

2 63 | ********

3 10 | *

4 11 | *

5 6 |

6 1 |

+-----------+---------+---------+-----------+

0 71.3 142.5 213.8 285.0

Variable 18

Value Freq

|

0 216 | ****************************************

1 73 | *************

2 97 | *****************

3 28 | *****

4 36 | ******

5 4 |

6 5 |

+-----------+---------+---------+-----------+

0 54.0 108.0 162.0 216.0

Variable 19

Value Freq

|

0 357 | ****************************************

1 86 | *********

2 4 |

3 2 |

4 4 |

5 3 |

6 3 |

+-----------+---------+---------+-----------+

0 89.3 178.5 267.8 357.0

Variable 20

Value Freq

|

0 391 | ****************************************

1 58 | *****

2 4 |

3 3 |

4 2 |

5 0 |

6 1 |

+-----------+---------+---------+-----------+

0 97.8 195.5 293.3 391.0

Variable 21

Value Freq

|

0 348 | ****************************************

1 57 | ******

2 27 | ***

3 5 |

4 17 | *

5 3 |

6 2 |

+-----------+---------+---------+-----------+

0 87.0 174.0 261.0 348.0

Variable 22

Value Freq

|

0 378 | ****************************************

1 38 | ****

2 26 | **

3 4 |

4 8 |

5 3 |

6 2 |

+-----------+---------+---------+-----------+

0 94.5 189.0 283.5 378.0

Variable 23

Value Freq

|

0 332 | ****************************************

1 44 | *****

2 45 | *****

3 14 | *

4 15 | *

5 4 |

6 5 |

+-----------+---------+---------+-----------+

0 83.0 166.0 249.0 332.0

Variable 24

Value Freq

|

0 313 | ****************************************

1 58 | *******

2 42 | *****

3 16 | **

4 22 | **

5 3 |

6 5 |

+-----------+---------+---------+-----------+

0 78.3 156.5 234.8 313.0

Variable 25

Value Freq

|

0 342 | ****************************************

1 37 | ****

2 45 | *****

3 15 | *

4 12 | *

5 4 |

6 4 |

+-----------+---------+---------+-----------+

0 85.5 171.0 256.5 342.0

Variable 26

Value Freq

|

0 212 | ****************************************

1 66 | ************

2 73 | *************

3 24 | ****

4 24 | ****

5 21 | ***

6 39 | *******

+-----------+---------+---------+-----------+

0 53.0 106.0 159.0 212.0

Variable 27

Value Freq

|

0 227 | ****************************************

1 92 | ****************

2 55 | *********

3 22 | ***

4 27 | ****

5 19 | ***

6 17 | **

+-----------+---------+---------+-----------+

0 56.8 113.5 170.3 227.0

Variable 28

Value Freq

|

0 304 | ****************************************

1 72 | *********

2 33 | ****

3 20 | **

4 16 | **

5 7 |

6 7 |

+-----------+---------+---------+-----------+

0 76.0 152.0 228.0 304.0

Variable 29

Value Freq

|

0 265 | ****************************************

1 56 | ********

2 60 | *********

3 19 | **

4 32 | ****

5 13 | *

6 14 | **

+-----------+---------+---------+-----------+

0 66.3 132.5 198.8 265.0

Variable 30

Value Freq

|

0 159 | ****************************************

1 97 | ************************

2 66 | ****************

3 49 | ************

4 35 | ********

5 27 | ******

6 26 | ******

+-----------+---------+---------+-----------+

0 39.8 79.5 119.3 159.0

--------------------------------------------------------------------------------

MULTIVARIATE DESCRIPTIVES

Analysis of the Mardia's (1970) multivariate asymmetry skewness and kurtosis.

Coefficient Statistic df P

Skewness 300.850 23015.015 4960 1.0000

SKewness corrected for small sample 300.850 23175.191 4960 1.0000

Kurtosis 1476.086 126.168 0.0000**

** Significant at 0.05

--------------------------------------------------------------------------------

STANDARIZED VARIANCE / COVARIANCE MATRIX (POLYCHORIC CORRELATION)

(Polychoric algorithm: Bayes modal estimation; Choi, Kim, Chen, & Dannels, 2011)

Variable 1 2 3 4 5 6 7 8 9 10 11 12 13 14 15 16 17 18 19 20 21 22 23 24 25 26 27 28 29 30

V 1 1.000

V 2 0.380 1.000

V 3 0.461 0.655 1.000

V 4 0.381 0.583 0.683 1.000

V 5 0.259 0.425 0.414 0.428 1.000

V 6 0.420 0.718 0.741 0.666 0.553 1.000

V 7 0.440 0.593 0.667 0.585 0.435 0.686 1.000

V 8 0.326 0.552 0.615 0.581 0.494 0.687 0.748 1.000

V 9 0.384 0.548 0.574 0.528 0.507 0.660 0.650 0.648 1.000

V 10 0.352 0.566 0.613 0.570 0.465 0.667 0.687 0.648 0.738 1.000

V 11 0.300 0.357 0.370 0.351 0.451 0.440 0.376 0.390 0.478 0.394 1.000

V 12 0.351 0.540 0.524 0.514 0.433 0.629 0.634 0.577 0.589 0.709 0.525 1.000

V 13 0.207 0.272 0.246 0.364 0.453 0.353 0.336 0.352 0.396 0.328 0.460 0.414 1.000

V 14 0.249 0.286 0.321 0.362 0.469 0.427 0.410 0.427 0.454 0.428 0.513 0.493 0.744 1.000

V 15 0.326 0.408 0.467 0.423 0.263 0.475 0.452 0.333 0.395 0.423 0.257 0.420 0.192 0.214 1.000

V 16 0.366 0.471 0.489 0.555 0.499 0.566 0.604 0.577 0.525 0.570 0.356 0.539 0.431 0.430 0.528 1.000

V 17 0.319 0.368 0.397 0.496 0.478 0.490 0.565 0.519 0.512 0.561 0.374 0.536 0.519 0.530 0.374 0.683 1.000

V 18 0.354 0.285 0.321 0.356 0.288 0.355 0.318 0.285 0.330 0.293 0.259 0.312 0.305 0.289 0.247 0.338 0.399 1.000

V 19 -0.002 -0.004 -0.008 0.054 0.309 -0.005 0.020 0.122 0.100 0.087 0.098 -0.012 0.277 0.231 -0.080 0.134 0.166 0.118 1.000

V 20 0.149 0.222 0.245 0.328 0.445 0.238 0.248 0.322 0.330 0.324 0.289 0.274 0.396 0.362 0.109 0.352 0.372 0.311 0.716 1.000

V 21 0.266 0.402 0.400 0.387 0.459 0.447 0.617 0.562 0.512 0.569 0.365 0.480 0.416 0.491 0.334 0.568 0.551 0.323 0.360 0.529 1.000

V 22 0.184 0.303 0.263 0.310 0.414 0.389 0.422 0.500 0.477 0.430 0.364 0.411 0.433 0.496 0.192 0.507 0.532 0.286 0.499 0.637 0.768 1.000

V 23 0.206 0.356 0.338 0.294 0.464 0.382 0.451 0.498 0.384 0.393 0.355 0.412 0.323 0.457 0.249 0.476 0.489 0.308 0.431 0.525 0.655 0.717 1.000

V 24 0.287 0.428 0.418 0.383 0.387 0.515 0.541 0.496 0.450 0.485 0.297 0.471 0.314 0.381 0.410 0.519 0.460 0.380 0.122 0.353 0.566 0.502 0.712 1.000

V 25 0.136 0.336 0.306 0.416 0.445 0.390 0.462 0.492 0.424 0.391 0.324 0.393 0.505 0.473 0.207 0.503 0.480 0.210 0.372 0.533 0.692 0.649 0.607 0.504 1.000

V 26 0.258 0.498 0.549 0.463 0.382 0.552 0.569 0.554 0.497 0.520 0.300 0.377 0.228 0.233 0.303 0.421 0.376 0.220 0.071 0.326 0.513 0.416 0.481 0.531 0.542 1.000

V 27 0.301 0.513 0.505 0.448 0.455 0.556 0.591 0.595 0.558 0.551 0.337 0.500 0.380 0.309 0.347 0.515 0.456 0.267 0.082 0.417 0.531 0.516 0.510 0.577 0.506 0.691 1.000

V 28 0.268 0.400 0.449 0.453 0.467 0.533 0.493 0.451 0.520 0.494 0.365 0.450 0.431 0.407 0.351 0.529 0.465 0.303 0.168 0.374 0.545 0.523 0.491 0.550 0.596 0.620 0.620 1.000

V 29 0.211 0.392 0.437 0.422 0.398 0.467 0.529 0.497 0.450 0.487 0.292 0.454 0.364 0.416 0.285 0.518 0.504 0.259 0.229 0.456 0.595 0.532 0.580 0.611 0.640 0.582 0.557 0.552 1.000

V 30 0.319 0.584 0.576 0.533 0.451 0.644 0.580 0.574 0.522 0.578 0.366 0.488 0.215 0.236 0.505 0.535 0.469 0.287 0.039 0.249 0.433 0.327 0.415 0.524 0.415 0.630 0.627 0.532 0.529 1.000

--------------------------------------------------------------------------------

ADEQUACY OF THE CORRELATION MATRIX

Determinant of the matrix = 0.000000106361367

Bartlett's statistic = 7179.9 (df = 435; P = 0.000010)

Kaiser-Meyer-Olkin (KMO) test = 0.92233 (very good)

BC Bootstrap 95% confidence interval of KMO = ( 0.920 0.921)

--------------------------------------------------------------------------------

EXPLAINED VARIANCE BASED ON EIGENVALUES

Variable Eigenvalue Proportion of Cumulative Proportion

Variance of Variance

1 13.76485 0.45883 0.45883

2 2.80759 0.09359 0.55241

3 1.58990 0.05300

4 1.07328 0.03578

5 1.03069 0.03436

6 0.85243 0.02841

7 0.79363 0.02645

8 0.72439 0.02415

9 0.64734 0.02158

10 0.61830 0.02061

11 0.57003 0.01900

12 0.50054 0.01668

13 0.48456 0.01615

14 0.44812 0.01494

15 0.42790 0.01426

16 0.39453 0.01315

17 0.36703 0.01223

18 0.33390 0.01113

19 0.32681 0.01089

20 0.30308 0.01010

21 0.28373 0.00946

22 0.25507 0.00850

23 0.24294 0.00810

24 0.22143 0.00738

25 0.20688 0.00690

26 0.18599 0.00620

27 0.17502 0.00583

28 0.15058 0.00502

29 0.11612 0.00387

30 0.10330 0.00344

--------------------------------------------------------------------------------

PARALLEL ANALYSIS (PA) BASED ON MINIMUM RANK FACTOR ANALYSIS

(Timmerman & Lorenzo-Seva, 2011)

Implementation details:

Correlation matrices analized: Polychoric correlation matrices

Number of random correlation matrices: 500

Method to obtain random correlation matrices: Permutation of the raw data (Buja & Eyuboglu, 1992)

Variable Real-data Mean of random 95 percentile of random

% of variance % of variance % of variance

1 47.9* 6.7 7.3

2 9.7* 6.3 6.8

3 5.3 6.0 6.4

4 3.6 5.8 6.2

5 3.4 5.5 5.9

6 2.8 5.3 5.6

7 2.7 5.1 5.4

8 2.4 4.9 5.1

9 2.2 4.7 4.9

10 2.0 4.5 4.7

11 1.8 4.3 4.5

12 1.7 4.1 4.3

13 1.5 3.9 4.1

14 1.4 3.6 3.9

15 1.3 3.4 3.6

16 1.2 3.2 3.4

17 1.1 3.0 3.3

18 1.0 2.8 3.1

19 1.0 2.6 2.9

20 1.0 2.4 2.7

21 0.9 2.2 2.5

22 0.8 2.0 2.3

23 0.8 1.8 2.1

24 0.7 1.6 1.8

25 0.5 1.3 1.6

26 0.5 1.1 1.4

27 0.4 0.9 1.2

28 0.3 0.6 0.9

29 0.1 0.4 0.6

30 0.0 0.0 0.0

* Advised number of dimensions: 2

--------------------------------------------------------------------------------

ROBUST GOODNESS OF FIT STATISTICS

Root Mean Square Error of Approximation (RMSEA) = 0.088; BC Bootstrap 95% confidence interval = ( 0.0830 0.0887)

Estimated Non-Centrality Parameter (NCP) = 430.520

Degrees of Freedom = 376

Test of Approximate Fit

H0 : RMSEA < 0.05; P = 1.000

Minimum Fit Function Chi Square with 376 degrees of freedom = 798.374 (P = 0.000010)

Robust Mean-Scaled Chi Square with 376 degrees of freedom = 1714.335 (P = 0.000010)

Chi-Square for independence model with 435 degrees of freedom = 55819.572

Non-Normed Fit Index (NNFI; Tucker & Lewis) = 0.972; BC Bootstrap 95% confidence interval = ( 0.969 0.978)

Comparative Fit Index (CFI) = 0.976; BC Bootstrap 95% confidence interval = ( 0.973 0.981)

Schwarz’s Bayesian Information Criterion (BIC) = 2265.950; BC Bootstrap 95% confidence interval = (2112.721 2281.385)

Goodness of Fit Index (GFI) = 1.000; BC Bootstrap 95% confidence interval = ( 1.000 1.000)

Adjusted Goodness of Fit Index (AGFI) = 1.000; BC Bootstrap 95% confidence interval = ( 1.000 1.000)

Goodness of Fit Index without diagonal values (GFI) = 1.000; BC Bootstrap 95% confidence interval = ( 1.000 1.000)

Adjusted Goodness of Fit Index without diagonal values(AGFI) = 1.000; BC Bootstrap 95% confidence interval = ( 1.000 1.000)

EIGENVALUES OF THE REDUCED CORRELATION MATRIX

Variable Eigenvalue

1 13.335135647

2 2.398250449

3 1.050694314

4 0.598347302

5 0.437733130

6 0.419396246

7 0.284013207

8 0.215137654

9 0.153562039

10 0.110915005

11 0.095181746

12 0.037950582

13 0.017964449

14 -0.011114473

15 -0.055873948

16 -0.060148892

17 -0.066407700

18 -0.080888195

19 -0.096541840

20 -0.127593925

21 -0.158084974

22 -0.173318049

23 -0.180226689

24 -0.210301084

25 -0.236904351

26 -0.256135084

27 -0.284000838

28 -0.290922625

29 -0.313370651

30 -0.396438587

--------------------------------------------------------------------------------

UNROTATED LOADING MATRIX

Variable F 1 F 2 Communality

V 1 0.450 -0.212 0.247

V 2 0.698 -0.320 0.590

V 3 0.745 -0.384 0.702

V 4 0.705 -0.241 0.555

V 5 0.632 0.116 0.413

V 6 0.831 -0.325 0.796

V 7 0.814 -0.199 0.703

V 8 0.786 -0.123 0.633

V 9a 0.765 -0.139 0.605

V 9b 0.791 -0.184 0.659

V 10a 0.541 0.038 0.294

V 10b 0.728 -0.144 0.551

V 11a 0.552 0.309 0.401

V 11b 0.602 0.268 0.434

V 12 0.517 -0.254 0.332

V 13 0.743 0.005 0.552

V 14 0.702 0.108 0.504

V 15 0.439 0.019 0.193

V 16a 0.224 0.664 0.491

V 16b 0.512 0.566 0.582

V 17 0.759 0.353 0.701

V 18 0.696 0.533 0.768

V 19 0.691 0.418 0.652

V 20 0.695 0.139 0.503

V 21 0.692 0.406 0.645

V 22 0.704 -0.038 0.497

V 23 0.742 0.006 0.551

V 24 0.702 0.115 0.506

V 25 0.703 0.204 0.536

V 26 0.718 -0.208 0.559

--------------------------------------------------------------------------------

ROTATED LOADING MATRIX

Variable F 1 F 2

V 1 0.530 -0.075

V 2 0.815 -0.108

V 3 0.906 -0.162

V 4 0.754 -0.019

V 5 0.396 0.347

V 6 0.923 -0.067

V 7 0.804 0.065

V 8 0.717 0.139

V 9 0.715 0.114

V 10 0.773 0.073

V 11 0.391 0.230

V 12 0.691 0.096

V 13 0.532 0.170

V 14 0.503 0.244

V 15 0.619 -0.098

V 16 0.576 0.265

V 17 0.457 0.363

V 18 0.327 0.174

V 19 -0.386 0.806

V 20 -0.078 0.799

V 21 0.295 0.651

V 22 0.093 0.827

V 23 0.187 0.699

V 24 0.394 0.426

V 25 0.197 0.687

V 26 0.582 0.203

V 27 0.574 0.266

V 28 0.371 0.451

V 29 0.377 0.469

V 30 0.736 0.023

ROTATED LOADING MATRIX

(loadings lower than absolute 0.300 omitted)

Variable F 1 F 2

V 1 0.530

V 2 0.815

V 3 0.906

V 4 0.754

V 5 0.396 0.347

V 6 0.923

V 7 0.804

V 8 0.717

V 9 0.715

V 10 0.773

V 11 0.391

V 12 0.691

V 13 0.532

V 14 0.503

V 15 0.619

V 16 0.576

V 17 0.457 0.363

V 18 0.327

V 19 -0.386 0.806

V 20 0.799

V 21 0.651

V 22 0.827

V 23 0.699

V 24 0.394 0.426

V 25 0.687

V 26 0.582

V 27 0.574

V 28 0.371 0.451

V 29 0.377 0.469

V 30 0.736

EXPLAINED VARIANCE OF ROTATED FACTORS AND RELIABILITY OF PHI-INFORMATION OBLIQUE EAP SCORES

Ferrando & Lorenzo-Seva (2016)

Factor Variance ORION (Overall Reliability of fully-Informative prior Oblique N-EAP scores)

1 10.274 0.948

2 5.882 0.819

The appropriate implementation of EAP score estimation in factor model involves to obtain point estimates that make use of the full prior information (in particular the inter-factor correlation matrix), and to complement the point estimates with measures of the reliability of these estimates. In order to achieve it, FACTOR computes: (1) the EAP score estimation named

'Fully-Informative Prior Oblique EAP scores'; and (2) the reliability estimates named ORION

(acronim for 'Overall Reliability of fully-Informative prior Oblique N-EAP scores').

See Ferrando & Lorenzo-Seva (2016) for further details.

--------------------------------------------------------------------------------

INTER-FACTORS CORRELATION MATRIX

Factor F 1 F 2

F 1 1.000

F 2 0.494 1.000

--------------------------------------------------------------------------------

STRUCTURE MATRIX

Variable F 1 F 2

V 1 0.493 0.187

V 2 0.762 0.295

V 3 0.826 0.286

V 4 0.745 0.354

V 5 0.567 0.543

V 6 0.890 0.389

V 7 0.837 0.463

V 8 0.786 0.494

V 9 0.771 0.467

V 10 0.809 0.455

V 11 0.504 0.423

V 12 0.738 0.437

V 13 0.616 0.433

V 14 0.624 0.493

V 15 0.570 0.207

V 16 0.706 0.549

V 17 0.636 0.589

V 18 0.413 0.335

V 19 0.013 0.616

V 20 0.317 0.760

V 21 0.617 0.797

V 22 0.502 0.873

V 23 0.532 0.791

V 24 0.605 0.621

V 25 0.537 0.784

V 26 0.682 0.491

V 27 0.705 0.549

V 28 0.594 0.634

V 29 0.608 0.655

V 30 0.747 0.386

--------------------------------------------------------------------------------

BIAS-CORRECTED AND ACCELERATED (BCa) BOOTSTRAP 95% CONFIDENCE INTERVALS FOR LOADING VALUES

Variable F 1 BCa Confidence Interval

V 1 0.530 ( 0.406 0.620)

V 2 0.815 ( 0.758 0.887)

V 3 0.906 ( 0.853 0.964)

V 4 0.754 ( 0.694 0.840)

V 5 0.396 ( 0.293 0.478)

V 6 0.923 ( 0.878 0.971)

V 7 0.804 ( 0.748 0.860)

V 8 0.717 ( 0.633 0.786)

V 9 0.715 ( 0.631 0.782)

V 10 0.773 ( 0.699 0.837)

V 11 0.391 ( 0.261 0.495)

V 12 0.691 ( 0.611 0.763)

V 13 0.532 ( 0.402 0.700)

V 14 0.503 ( 0.370 0.638)

V 15 0.619 ( 0.512 0.696)

V 16 0.576 ( 0.479 0.661)

V 17 0.457 ( 0.372 0.556)

V 18 0.327 ( 0.195 0.428)

V 19 -0.386 ( -0.533 -0.267)

V 20 -0.078 ( -0.224 0.021)

V 21 0.295 ( 0.209 0.388)

V 22 0.093 ( 0.001 0.172)

V 23 0.187 ( 0.085 0.285)

V 24 0.394 ( 0.300 0.526)

V 25 0.197 ( 0.108 0.299)

V 26 0.582 ( 0.473 0.688)

V 27 0.574 ( 0.465 0.662)

V 28 0.371 ( 0.265 0.465)

V 29 0.377 ( 0.284 0.481)

V 30 0.736 ( 0.659 0.830)

Variable F 2 BCa Confidence Interval

V 1 -0.075 ( -0.168 0.033)

V 2 -0.108 ( -0.206 -0.047)

V 3 -0.162 ( -0.241 -0.097)

V 4 -0.019 ( -0.108 0.058)

V 5 0.347 ( 0.254 0.451)

V 6 -0.067 ( -0.143 -0.006)

V 7 0.065 ( -0.012 0.123)

V 8 0.139 ( 0.062 0.227)

V 9 0.114 ( 0.037 0.207)

V 10 0.073 ( 0.006 0.151)

V 11 0.230 ( 0.102 0.349)

V 12 0.096 ( 0.008 0.184)

V 13 0.170 ( 0.013 0.293)

V 14 0.244 ( 0.113 0.362)

V 15 -0.098 ( -0.172 0.011)

V 16 0.265 ( 0.164 0.359)

V 17 0.363 ( 0.245 0.450)

V 18 0.174 ( 0.068 0.287)

V 19 0.806 ( 0.693 0.939)

V 20 0.799 ( 0.699 0.902)

V 21 0.651 ( 0.548 0.722)

V 22 0.827 ( 0.752 0.899)

V 23 0.699 ( 0.586 0.780)

V 24 0.426 ( 0.313 0.524)

V 25 0.687 ( 0.594 0.768)

V 26 0.203 ( 0.084 0.332)

V 27 0.266 ( 0.147 0.385)

V 28 0.451 ( 0337 0.549)

V 29 0.469 ( 0.368 0.563)

V 30 0.023 ( -0.095 0.114)

--------------------------------------------------------------------------------

INDICES OF FACTOR SIMPLICITY

Bentler (1977) & Lorenzo-Seva (2003)

Bentler's simplicity index (S) = 0.97891 (Percentile 100)

BC Bootstrap 95% confidence interval = ( 0.967 0.986)

Loading simplicity index (LS) = 0.36385 (Percentile 99)

BC Bootstrap 95% confidence interval = ( 0.293 0.421)

--------------------------------------------------------------------------------

BIAS-CORRECTED BOOTSTRAP 95% CONFIDENCE INTERVALS FOR INTER-FACTORS CORRELATION VALUES

1 -- 2 0.494* ( 0.437 0.561)

* Significantly different from zero at population

--------------------------------------------------------------------------------
